# The Long-Term Effect of Calcium Hydroxide, Calcium-Enriched Mixture Cement and Mineral Trioxide Aggregate on Dentin Strength

**Published:** 2014-07-05

**Authors:** Fariborz Moazami, Safoora Sahebi, Davoud Jamshidi, Aliasghar Alavi

**Affiliations:** a Department of Endodontics, Dental School, Shiraz University of Medical Sciences, Shiraz, Iran; b Department of Endodontics, Dental School, Gazvin University of Medical Sciences, Gazvin, Iran; c Biomaterials Research Center, Dental School, Shiraz University of Medical Sciences, Shiraz, Iran

**Keywords:** Calcium-Enriched Mixture Cement, Calcium Hydroxide, CEM, Flexural Strength, Fracture Resistance, Mineral Trioxide Aggregate, MTA, Root Canal Filling Materials

## Abstract

**Introduction:** Many of highly-alkaline dental materials have some adverse effects on physical properties of dentin. As basic substances, mineral trioxide aggregate (MTA), calcium hydroxide (CH) and the new endodontic material, calcium-enriched mixture (CEM) cement, may adversely affect dentin. The purpose of this *in vitro* study was to evaluate the effect of long-term application of CEM cement, MTA and CH on flexural strength of bovine dentin. **Materials and Methods:** Three hundred and twenty bovine dentin samples were divided into 4 groups, which were either exposed to CEM cement, CH, MTA or normal saline (control group). Samples of each group were divided into 4 subgroups which were tested by means of Instron Universal Testing Machine for periods of 7, 30, 180 and 365 days after exposure to the test materials. The required force for sample breakage was recorded. The data were analyzed by the two-way ANOVA and Tukey tests. **Results:** The mean value of forces to break the samples in CEM cement and CH groups was significantly lower than the control group after 1 month (*P*<0.05). After 180 days, the samples of CEM cement group retrieved their strength but in MTA and CH groups the time interval weakened the samples. After one year of exposure to CH and MTA, flexural strength of the dentin reduced to 72% and 38.7%, respectively (*P*<0.05). Yet the flexural strength of samples in CEM cement group did not change significantly compared to control group. **Conclusion:** Following 365 days of application of experimental materials to bovine dentin, the CEM cement showed an interesting result and the samples in this group reached their initial strength during the first week of the study but the other materials caused a reduction in dentin strength at the end of the study.

## Introduction

Root canal treatment (RCT) is a common dental procedure to retain teeth with necrotic or infected pulps. One of the most common clinical problems affecting the endodontically treated teeth is fracture, which can potentially lead to tooth extraction [1]. Loss of tooth structure due to reparation of the access cavity and caries removal, make these teeth more susceptible to fracture [2, 3].

Irrigants, medicaments or root canal filling materials may also affect the mechanical properties of dentin and make it more fragile [4]. Some materials can cause dentin erosion and softening during endodontic treatment. These changes make the dentin and root structure more vulnerable to fracture [5, 6].

Since its introduction to dentistry by Hermann in 1920, calcium hydroxide (CH) has been commonly used as an intra-canal inter-appointment medicament. Various biological properties have been attributed to this substance, such as antimicrobial activity, tissue-dissolving ability, inhibition of tooth resorption, and induction of hard tissue formation [7]. In aqueous solution, CH releases hydroxyl ions and produces a pH of 12.4, which is the antibacterial mechanism of this biomaterial [8]. Researchers found that using CH as an intra-canal medicament can weaken the dentin compared to non treated teeth. They suggested that CH may affect the dentin structure by reducing its organic component, which may influence the mechanical properties of dentin [9-11]. 

**Table 1:** Mean (SD) of flexural strength in 16 groups

**Time**	**Normal Saline**	**CH**	**MTA**	**CEM**
**7 days**	144.6 (35.5)	143.6 (36.5)	155.7 (56)	140.6 (48.9)
**30 days**	152.9 (52.4)	110.7 (38.8)	136.2 (34.2)	109.07 (33.4)
**180 days**	129.2 (54.2)	70.8 (24.5)	103.8 (38.8)	133.7 (49)
**365 days**	152.8 (60.1)	42.2 (13.9)	93.6 (37)	125 (38.5)

Mineral trioxide aggregate (MTA) was first introduced in 1993 and was approved for endodontic application in 1998 [12, 13]. Portland cement is the major component of MTA and bismuth oxide has been added for its radiopacity [14]. MTA produces an initial pH of 10.2, which rises to 12.5 during the next three hours [15]. MTA is used as a material for root-end filling, direct pulp capping, repair of root and furcation perforations, and also apexification [16]. MTA has also shown a weakening effect on dentin, probably due to breakdown of the protein structure, caused by its alkalinity [17].

A new endodontic cement, named calcium-enriched mixture (CEM) cement, has been recently developed with different chemical composition from MTA but with similar clinical applications. This material can produce hydroxyapatite crystals from endogenous and exogenous ions sources. This cement has the similar biocompatibility to that of MTA, with more efficient properties such as good handling characteristics, shorter setting time, and no tooth staining [18]. CEM cement has acceptable film thickness, flow and sealing ability compared to MTA [19, 20]. Also, it has lower cytotoxicity on different cell lines [21, 22]. The antibacterial effect of CEM cement is comparable to CH and greater than MTA [23]. CEM cement produces favorable results when used as a root-end filling material [24] and a pulp capping agent in primary and permanent teeth [25, 26]. It appears that CEM cement can be used as a pulpotomy material in permanent teeth with open apices [27] and also in mature and primary teeth [28, 29]. This cement can be used for management of internal root resorption and repair of furcal perforation [30, 31].

As this material is used for endodontic treatment, the effect of CEM cement on the mechanical properties of teeth substrates during its application could help clinicians improve their clinical procedure. The aim of this *in vitro* study was to evaluate the effect of CH, MTA and CEM cement on the flexural strength of standardized dentin bars during different time periods.

## Methods and Materials

The dentin specimens were prepared as originally described by Haapasalo and Ørstavik [32]. Intact bovine incisors were used for this experiment. Less than one week prior to commencing the study, the teeth were kept in physiologic saline to prevent their dehydration. The apical 5-mm and two-thirds of the crowns were removed with a water-cooled, high-speed diamond bur (D&Z, Wiesbaden, Germany). Samples were embedded in a self-cure acrylic resin, and a 3.5-mm diameter twist drill was used parallel to the long axis of root canal to widen the canal. Symmetrical cylinders of dentin with outer and inner diameter of approximately 6- and 3.5-mm, respectively and with length of 15-mm was prepared with this method. These cylinders were cut lengthwise into four symmetrical pieces using a diamond disk (Leco, St. Joseph, MI, USA).

A cross-section cut was made in order to prepare four 10-mm-long pieces. All sections were then weighted on a Mettler balance (Mettler Instrument Company, Hightstown, NJ, U.S.A.) to verify similarity of slices for use in this study. Samples that were out of the normal range were discarded. The remaining samples (*n*=320) were randomly divided to 16 groups with 20 samples each. The samples were placed into the Petri dishes and on the surfaces of test materials with their dentinal side in contact with the material, as follows:

1. Four Petri dishes containing a 2-mm-thick bulk of CH [calcium hydroxide powder (Merck, Darmstadt, Germany)] which was mixed with distilled water to make a creamy paste.
2. Four Petri dishes containing 2-mm-thick bulk of CEM cement (BioniqueDent, Tehran, Iran) which was prepared according to the manufacturer instructions.
3. Four Petri dishes containing 2-mm-thick bulk of MTA (Angelus, Lodrina, Brazil) which was also prepared according to the instructions provided by the manufacturer.
4. Four Petri dishes containing physiologic saline with 2-mm depth. This group was considered as a negative control group.

The samples remained in the dishes for 7, and 30, 180 and 365 days. Distilled water was added to the Petri dishes every 3 to 4 days as needed to maintain their moisture. All Petri dishes were stored at 37^°^ C and 100% humidity.

Upon completion of each time period, each sample was rinsed with tap water. The dentin bars were subjected to the three-point bend test, using an Instron Universal Testing Machine (Z010, Zwick GmbH, Ulm, Germany). Each bar was loaded at the mid-point through the loading head and shaft. The loaded testing machine was set at a speed of 1 mm/min. The required force for breaking each sample was recorded in MPa.

**Figure 1:**
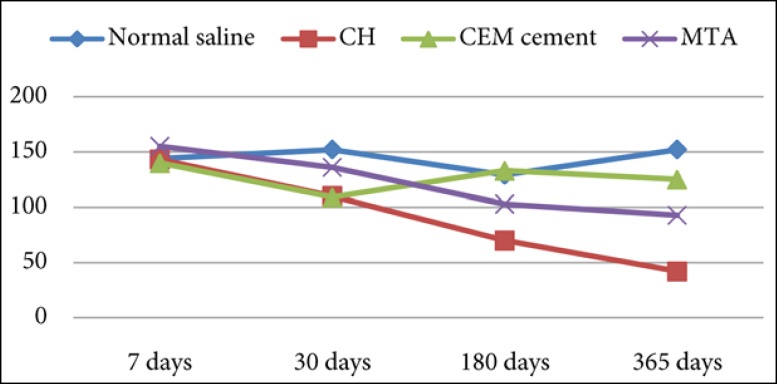
Flexural strength means (MPa) for each group

***Statistical analysis***

The raw data were tabulated and the means and standard deviation was calculated for each group. The results were analyzed using the two-way ANOVA with material type and time, as two factors, and flexural strength as a response variable. If a significant interaction was identified, the one-way ANOVA test was performed for each time-duration. Data were analyzed using the one-way ANOVA test for comparison of the groups as a whole, and the Tukey’s post-hoc test was used to compare the groups with each other. Statistical calculations were performed with the SPSS software (SPSS INC., Chicago, IL, USA, version 16). A *P*-value less than 0.05 was considered statistically significant.

## Results

Table 1 and Figure 1 summarize the result of the fracture strength testing. The force required to fracture dentin samples in each group was different, depending on time interval.

The materials were compared with each other at four time periods: 7, 30, 180 and 365 days. The differences between the amounts of fracture load was not statistically significant for any of the 7-day specimens (*P*>0.05).

There was a significant reduction in flexural strength in CEM cement and CH groups after 30 days in comparison with normal saline group, which was 28% and 27.5%, respectively (*P*<0.05).

The decrease in mean value of flexural strength after 180 days in CH and MTA groups was more than 45% and 19%, respectively (*P*<0.05). Samples in CEM group retrieved their strength after 180 days and reached the strength amount slightly more than normal saline group.

After 365 days of exposure to CH and MTA, flexural strength of the dentin reduced to 72% and 38.7% ,respectively (*P*<0.05), but the flexural strength of samples in CEM cement group did not change significantly in comparison with the control group. The reduction of flexural strength in dentin bars in CEM group was less than MTA without any significant differences (*P*>0.05).

## Discussion

This study revealed the alteration of dentin physical properties after 30-day exposure to CH, MTA and CEM cement. All the samples showed a decrease in their strength in comparison with normal saline group. This finding is in agreement with the other studies that applied these materials on dentin surfaces for ±30 days [11, 33-35].

The current study used bovine incisors since they were easy to obtain in a good condition and showed less variability in comparison with human teeth [36]. No significant differences were found in diffusion rates of CH paste in human and bovine teeth [37]. Although bovine and human teeth have some differences in their morphology, several studies have demonstrated similarities between their substrates. Sano *et al*. [38] showed the same ultimate tensile strength and modulus of elasticity (MOE) between human and bovine dentin. Moreover, other studies have also reported similar results in bonding tests and the number and distribution of dentinal tubules between bovine and human dentin [33, 36, 39]. Above all, bovine incisors show less variation in anatomical morphology than human teeth. Therefore, bovine teeth provide a standard and reproducible material that allows evaluation of different treatment modalities for management of this clinical situation.

Several studies have indicated that CH has a weakening effect on dentin [11, 17, 34, 35, 40], but such studies on the effect(s) of MTA on fracture resistance of teeth have reported contradictory results. The results of this study is not in agreement with those of Bortoluzzi *et al.* [41], Hatibovic-Kofman *et al. *[42] and Milani *et al.* [43], who found a significant increase in fracture strength of dentin when the teeth were filled with MTA. It seems that the main reason for this difference is in methodology of these studies. Those studies did not evaluate the pure effect of experimental materials on dentin strength, because the force was applied on root-canal filled teeth while in this study, the load was applied on dentin bars. 

A finite element analysis showed that the materials with similar MOE to dentin could reinforce the weak roots [44], also Cauwels *et al. *[45] showed that the samples obturated with gutta-percha, MTA and calcium-phosphate bone cement (CPC) had a significantly higher fracture resistance compared to unfilled samples. In our study, the samples were rinsed before testing to remove the remnants of experimental materials from dentin bars and also the force was applied on the standard dentin samples instead of root canal filled roots. Furthermore, they applied parallel forces or forces with 135^°^ angulation to the long axis of the tooth on the incisal edge but in the present study, 3-point bending load was applied on dentin bars. Methodology of this study was almost similar to the studies by White *et al.* [17] and Sahebi *et al.* [35]. Both of them showed that MTA decreased dentin strength.

The weakening effect of CH, CEM cement and MTA observed in this study maybe due to the break-down of protein structures caused by the alkalinity of the used materials. Previous studies have also reported that materials with high alkalinity can cause conformational changes in protein structure [17].

This study showed that after 365 days of exposure to CH and MTA, the samples become more fragile, but surprisingly the samples which were exposed to CEM cement almost retrieved their initial strength after six months of exposure and maintained their flexural strength till the end of the study.

This behavior of CEM cement, despite the fact that its initial pH is comparable to that of CH and MTA, is unclear but may be explained by the pH changes during the long-term contact of samples with CEM cement compared to CH and MTA. The pH changes of CEM cement during one month has been investigated in one study [46], but there is no available study regarding the alteration of pH caused by CEM cement in long-term studies. It seems that further studies are needed for better understanding of this phenomenon.

## Conclusion

In conclusion, it is highly probable that alkaline endodontic materials reduce the mechanical properties of root dentin; however CEM cement may be the most favorable biomaterial in this regard as it does not negatively affect the dentinal strength.
